# The Cost of Home Birth in the United States

**DOI:** 10.3390/ijerph181910361

**Published:** 2021-10-01

**Authors:** David A. Anderson, Gabrielle M. Gilkison

**Affiliations:** Department of Economics and Business, Centre College, Danville, KY 40422, USA; gabrielle.gilkison@centre.edu

**Keywords:** home birth, cost, global fee, midwife, midwifery, out-of-hospital birth, community birth

## Abstract

Policy decisions about the accessibility of home birth hinge on questions of safety and affordability. Families consider safety and cost along with the comfort and familiarity of birthing venues. A substantial literature addresses safety concerns, generally reporting that for low-risk mothers in the care of credentialed midwives, the safety of planned home births is comparable to that in birth centers and hospitals. The lack of notable safety tradeoffs for low-risk mothers elevates the relevance of the economic efficiency of home births. The available cost figures for home births are largely out of date or anecdotal. The purpose of this research is to offer scholars, policymakers, and families improved estimates of both the cost of home births and the potential savings from greater access to home births. On the basis of a nationwide study, we estimate that the average cost of a home birth in the United States is USD 4650, which is significantly below existing cost estimates for an uncomplicated birth center or hospital birth. Further, we find that each shift of one percent of births from hospitals to homes would represent an annual cost savings to society of at least USD 321 million.

## 1. Introduction

With the appreciable safety of home births substantiated by existing literature, e.g., [1,2,3,4,5], associated policy discussions can focus on economic efficiency. Legislators and insurers need credible estimates of the cost of home births to inform decisions about legality and accessibility. Available estimates for home births in the United States are generally outdated or rely on small samples with limited geographic representation. This study reports findings based on a representative sample of home birth fees collected from midwifery practices across the country. A comparison of the estimated costs of births in homes, birth centers, and hospitals demonstrates opportunities for savings on the most common reason for hospital admission in the United States. 

The limited popularity of home births has shown both vulnerability and promise in recent decades. The number of home births decreased in the United States from 1990 through 2004, overshadowed by hospital births as the predominant cultural norm [6]. That trend reversed in the early 2000s, and by 2015, the United States had the largest number of home births among the industrialized countries, 38,542 [7]. By 2017, 1 in 62 births occurred outside of hospitals [6].

As interest in home births continues to rise, the need for specific and readily available cost estimates also grows. The ability to compare prices for birth services encourages competition among hospitals, birth centers, and midwives, which can both increase the quality of care and decrease the cost [8]. Cost comparisons also allow growing families to find birthing options that fit their needs. On a broader level, estimates of the cost of home birth permit both federal and state legislators to examine policies on midwifery and access to out-of-hospital births. Past research indicates that policies more favorable to home births with credentialed midwives could achieve substantial savings, e.g., [9,10,11,12]. The accuracy of such findings rests on the quality of cost estimates for home births.

Up-to-date costs for home births across the United States are currently lacking. In a review of research on the cost of alternative birth settings from six countries including the United States, Scarf et al. [13] conclude that the cost of home birth is difficult to find and that more research is needed to meet growing demands. The present research aims to fill that void by providing information on the cost of home births for the benefit of scholars, legislators, insurance companies, and families among other interested parties.

Section 2 of this article reviews the literature on the cost of home, birth center, and hospital births. Section 3 explains the data collection methods for this research. Section 4 presents the findings. Section 5 discusses implications and applications. Section 6 concludes the article.

## 2. Literature Review

Anderson and Anderson [9] estimated the average cost of home birth in the United States based on survey data collected from 57 midwifery practices in 29 states. They reported an average cost of USD 3413 in 1991. A second data collection from 54 practices in 26 states in 1998 produced an average cost of USD 3039. These and all figures are adjusted for inflation using the Consumer Price Index [14] unless otherwise noted. Given the dearth of more recent research of this type, inflation-adjusted versions of the 1991 figure appear often in the literature, including in Daviss, Anderson, and Johnson [8], Janssen et al., [12], Johnson and Daviss [3], and Scarf et al., [13]. In a meta-analysis of home birth cost estimates, Press [15] lists only two studies from the United States: Anderson and Anderson [9], and Health Management Associates [11], which looked exclusively at data from Washington State.

The dated cost estimates in the literature force the online sources most accessible to data seekers to offer broad estimates, often with no clear basis. For instance, a Google search for “How much does a home birth cost in the United States?” yields a Parents.com estimate of USD 3000 to USD 9000 [16], a Parenting. Firstcry.com estimated average of USD 3000 to USD 6000 [17], and a MamaNatural.com estimate of USD 2000 to USD 4000 for midwifery care [18] among other estimated ranges. To illustrate the round numbers available online in mid-2021, these particular figures are not adjusted for inflation.

Estimates of the average cost of hospital and birth center births are more readily available. Johnson et al. [19] estimated a USD 13,562 average cost for a vaginal hospital birth in the United States. This is the sum of all facility, professional, and newborn fees, including payments by insurers and patients, for individuals with employer-provided insurance. Their data came from 35 states, and the costs ranged from USD 8321 in Arkansas to USD 19,459 in New York. The American Association of Birth Centers [20] estimated a USD 8309 total fee for a birth center birth using data from 120 birth centers across the United States. The costs reported in their survey ranged from USD 1811 to USD 18,399, and include professional fees, facility fees, and newborn fees.

## 3. Methodology

The survey instrument sent to midwifery practices collected information on whether the respondents had state licensure or registration and what type of national certification they had received, if any. It then asked for their charge for prenatal care, delivery, and postpartum care, and each available combination of those services, with and without insurance. The survey also requested the respondents’ city and state, and any additional information on the midwives’ charges that the investigators should take into consideration. 

To achieve broad geographic representation, we sent the survey to members of the midwifery communities in every state. Midwifery practices were initially identified using an internet search for “home birth” or “midwife” and the state name, just as a potential birthing person might do when researching options for birth care. We then pulled contact information from the providers’ websites. The survey was then distributed through direct email communication or through “Contact Us” links on the provider’s website.

The survey instrument was sent by email to 176 midwifery practices in all 50 states. After follow-up emails to every non-respondent and selective phone calls and data collection from the websites of non-respondents, the response rate was 32 percent. Some recipients of the email indicated that, although they were midwives, they did not assist with home births, making the response rate among practices that do assist with home births higher. A Google Forms version of the survey was also sent to members of the Big Push midwifery organization. The digital format of the Google Forms survey was user friendly on a variety of devices including smartphones and elicited 73 additional responses. All data collection was conducted in 2021.

## 4. Results

The findings are based on data obtained from 129 midwifery practices in 49 states, the exception being Nebraska, where home births are rare due to exceptional legal restrictions on midwives [21]. The data represent the cost of home births for an even larger number of midwives and locations because midwifery practices typically employ several midwives serving multiple communities and sometimes multiple states. 

Certified professional midwives made up 76.7 percent of the respondents, 13.2 percent were certified nurse midwives, and the remaining 10.1 percent were in the “other” category, having indicated that they are not nationally credentialed or having given a response that could not be categorized. The latter group included a small number of lay midwives whose training is typically through apprenticeships rather than graduate-level education.

A standard home birth package includes prenatal care, delivery, and postpartum care. The price of this package is described as the global fee. Based on the data collected, the average global fee for a home birth in the United States is USD 4650, with a median of USD 4400, a mode of USD 4000, and a standard deviation of USD 1413. The global fee ranged from USD 2000 to USD 9921. Only 24.0 percent of practices reported an option for delivery only. For those 31 practices, the mean delivery-only fee was USD 3777, with a range from USD 2000 to USD 6000, a median and mode of USD 4000, and a standard deviation of USD 1028.

Table 1 provides home birth fees for five regions of the United States. Data are broken down by region rather than by state so as not to reveal cost figures for particular practices in states with a small number of home birth providers. States were categorized into regions according to the National Geographic Society’s [22] designations.

Because the cost of home births varies considerably by region and state, it is relevant to provide an average global fee that is adjusted to reflect the larger populations in locations with relatively high birth fees. The population-weighted average global fee is calculated as the sum of each state’s proportion of U.S. residents multiplied by its average global fee for a home birth. The population-weighted average global fee for the United States is USD 5135. In the calculation of this figure, the fee assigned for Nebraska residents was the average of the fees in the six contiguous states. The assumption was that if growing acceptance of home births led Nebraska to legalize midwife-assisted home births, the fees would be similar to those in neighboring states. If Nebraska is taken out of the calculation, the population-weighted average global fee is a very similar USD 5138. In the comparisons with birth center and hospital births below, we use the unweighted average global fee because the fees for the other birth locations are also unweighted.

Insurance currently does not cover home births in many cases, which helps to explain why only 20.9 percent of practices reported a different fee for insured clients. Of the practices that did report a different fee, the average charge for insured clients was USD 5050, 8.6 percent higher than for uninsured clients. The fee for insured clients ranged from USD 2000 to USD 16,500 with a median of USD 4500, a mode of USD 4000, and a standard deviation of USD 2043. The somewhat higher fee for insured clients reflects those clients’ greater ability to pay and the insurance companies’ caps on reimbursement.

The average global fee increases with graduate-level training and is higher for certified nurse midwives than for certified professional midwives. Midwives in the “other” category that included lay midwives charged an average global fee of USD 4082. Among certified professional midwives, the average global fee was USD 4619. The certified nurse midwives charged an average of USD 5202.

## 5. Discussion

When comparing the costs of births in homes, birth centers, and hospitals, it should be noted that out-of-hospital birth packages may include prenatal and postpartum care beyond what is received with a hospital birth package [23,24]. The present study compares the average cost of a typical set of home birth and birth center services with the average cost of a typical set of hospital birth services. That captures the actual differences in cost, and although the services may differ, it is clear that an out-of-hospital birth and a hospital birth are not the same experience even disregarding the extent of prenatal and postpartum care. For that reason, in this study we focus on the cost of the typical package in each setting, acknowledging the inherent differences in care.

Given that all Medicaid and Health Insurance Marketplace plans cover childbirth [25], the potential savings from increased access to out-of-hospital births are relevant not only to growing families, but also to insurance providers, employers that fund health insurance, and everyone affected by federal and state budgets. Medicaid covers 42.1 percent of U.S. births [26], which indicates the appreciable benefits taxpayers could receive from lower birth costs. If the government is able to spend less on Medicaid-covered births due to an increase in home births, it can take any combination of three approaches: (1) the government can reduce its debt and the corresponding interest and debt payments funded by taxpayers; (2) it can keep government debt the same and lower tax collections for Medicaid; or (3) it can keep the debt and taxes the same and spend more on other government services such as infrastructure, national security, and public assistance programs. Each of these approaches is an improvement for taxpaying households.

For women with employer-covered health plans, the estimated average out-of-pocket maternity cost is USD 5238 for all births and USD 4945 for vaginal births [27]. Given that the out-of-pocket cost of a vaginal birth for women with insurance approximates the total cost of a midwife-assisted home birth, greater access to home births would clearly lower the financial burden on those with partial assistance from employer-provided insurance as well as those without it. Regarding the portion paid by insurance companies, it should be noted that the cost of insurance claims is ultimately covered by premiums paid by employers or individuals. It is unclear how a decrease in the cost of birth would be shared between insurance providers in the form of higher profits and insurance policyholders in the form of lower premiums, but both parties would stand to benefit from lower costs. 

The USD 8309 estimated average cost of a birth center birth [20] and the USD 4650 estimated average cost of a home birth fall below the USD 13,562 estimated average cost of a vaginal hospital birth [19] by USD 5252 and USD 8912, respectively. Anderson et al. [11] and Howell et al. [28] explain that the difference in vaginal birth costs is a conservative estimate of the savings from out-of-hospital births because there are also higher rates of cesarean births and low-birth-weight births among low-risk mothers who have planned hospital births in the United States.

**Figure 1:**
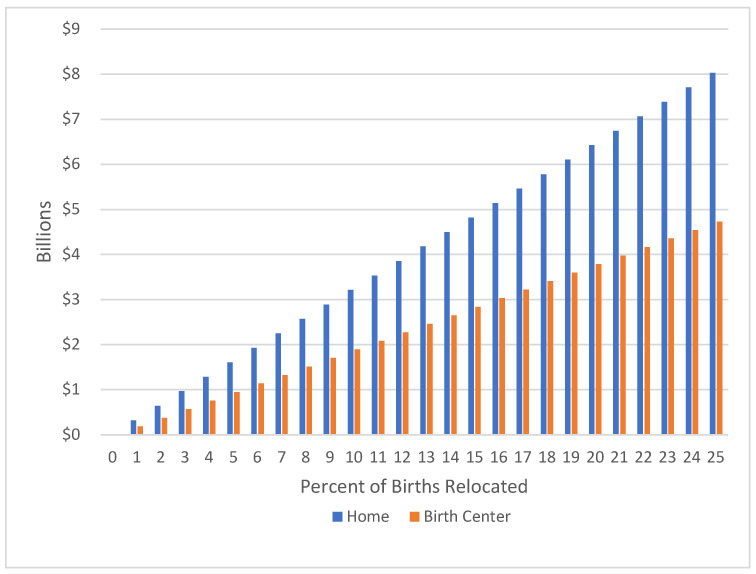
Potential Savings from Increases in the Percent of Out-of-Hospital Births.

In 1900, virtually all births were in homes [29]. In 2019, 1.0 percent of U.S. births were in homes, most often assisted by certified professional midwives, and 9.8 percent were assisted by certified nurse midwives, generally in birth centers, sometimes in homes or hospitals [26]. Changes in policies, information, and attitudes could change the access and popularity of home births as discussed below. Figure 1 indicates the additional savings for society if various percentages of U.S. births occurred in homes with credentialed midwives or in birth centers rather than in hospitals. Considering the 3,605,201 U.S. births in 2020 [30], each shift of one percent of births from hospitals to homes would represent a savings of USD 321 million on the global fee. The analogous shift from hospitals to birth centers would save USD 189 million. The savings would be USD 1.6 billion if an additional 5 percent of U.S. births shifted from hospitals to homes, and another USD 1.9 billion if 10 percent of U.S. births shifted from hospitals to birth centers. The Netherlands models the modern potential for home birth acceptance, with 13 percent of births being in homes, although that number is declining [31].

If greater access and acceptance shift a sizable proportion of births into homes and birth centers, market forces could further alter birth fees and the quality of care. Competition from the growing popularity of community birth could serve as a check on fees for hospital births, and on cesarean rates, prenatal and postpartum care, and associated outcomes such as low-birthweight births. At the same time, the global fees for midwife-assisted home births would be subject to related market forces. The supply of midwives is relatively elastic, both because they require fewer years of training than an obstetrician-gynecologist and because there is pent-up supply. Organizations of midwives are actively seeking to practice, or practice more, in states where laws constrain home births [32]. In the event that increases in the demand for home births do outpace increases in the supply of midwifery services, the result will be higher global fees for home births concurrent with lower fees for hospital births. Even so, the cost of childbirth will have decreased for the over 98 percent of families that would not deliver at home in the absence of change [33].

These findings are relevant to several ongoing policy decisions that affect home birth accessibility. More than three-quarters of the survey respondents were certified professional midwives (CPMs). CPMs are licensed to practice in 34 states and the District of Columbia [34]. The federal government does not recognize CPMs as Medicaid service providers, but 13 states have amended their plans to cover CPM services [35]. Certified nurse midwives, whose training includes nursing school along with graduate training in midwifery, are licensed in every state but are less likely to offer home births. An overview of the differences between these and other types of midwives is available from GraduateNursingEDU.org [36]. 

Some states place additional restrictions on home births. For example, 19 states require CNMs to establish a written agreement with a collaborating physician that specifies actions by the midwife that the physician must supervise [37]. Nebraska essentially prohibits midwives from assisting home births. That state recognizes only CNMs and not other types of midwives, requires CNMs to have a practice agreement with a physician, and expressly prohibits CNMs from attending a home delivery [21]. Coupled with the existing literature on the safety of home births for low-risk women, the specifics on potential cost savings from increased access to home births can now inform policy decisions at every level.

The data in this study are limited in that they do not capture all of the influences on the cost of births. Many midwife respondents indicated that they are willing to adjust their fees downward for clients with low incomes. Thus, the estimated home birth costs can be considered an upper bound on the actual fees charged for home births. Correspondingly, the estimated cost savings from increases in home births are conservative estimates of the actual cost savings that could be realized with greater access to home births. It should also be recognized that the focus of this study is the cost of births to society, and that the out-of-pocket costs to families depend on the share of birth costs covered by any available insurance coverage.

## 6. Conclusions

This study provides an estimate of the average cost of a midwife-assisted home birth in the United States. We also estimate the potential collective savings for families, insurers, and taxpayers from an increase in out-of-hospital births. Federal and state governments must decide whether to lift restrictions on midwives, while public and private insurers face the question of whether to cover home births. There are more than 3.6 million births every year in the United States. With the cost of birth spread across public assistance programs, employer-funded insurers, family-funded insurers, and families paying out-of-pocket, the 65.7% lower cost of home births relative to hospital births represents an opportunity for substantial savings for governments, employers, insurance providers, and households. Changes in legislation, corporate policies, and attitudes regarding out-of-hospital births could reduce the health care burden in the United States by several billion dollars annually.

## Figures and Tables

**Table 1:** U.S. Home Birth Fees by Region. (All fees are in USD.)

Region	N	Mean	Min	Max	Median	Mode	SD
West	21	4714	3500	8000	4500	3500	1037
Midwest	41	3976	2000	6050	4000	4500	936
Northeast	20	5299	2000	9000	5025	4800	1740
Southeast	32	4958	2000	9921	4350	4000	1702
Southwest	10	4805	2950	7500	4600	4200	1418
United States	129	4650	2000	9921	4400	4000	1413

## Data Availability

To protect the financial information of particular midwifery practices, the data are available at regional and national levels of aggregation as provided in the manuscript. Contact the corresponding author with requests for any other subsets of the data that would maintain the anonymity of individual practices in small states.
